# The impact of diagnostic criteria for gestational diabetes on its prevalence: a systematic review and meta-analysis

**DOI:** 10.1186/s13098-019-0406-1

**Published:** 2019-02-01

**Authors:** Samira Behboudi-Gandevani, Mina Amiri, Razieh Bidhendi Yarandi, Fahimeh Ramezani Tehrani

**Affiliations:** 1 Reproductive Endocrinology Research Center, Research Institute for Endocrine Sciences, Shahid Beheshti University of Medical Sciences, No 24, Parvane Street, Yaman Street, Velenjak, Tehran, P.O.Box: 19395-4763, Iran; 2 Department of Epidemiology and Biostatistics, School of Public Health, Tehran University of Medical Sciences, Poor sina street, Tehran, P.O.Box: 1417653761, Iran

**Keywords:** Diagnostic criteria, Gestational diabetes, Meta-analysis, Prevalence

## Abstract

**Background:**

The absence of universal gold standards for screening of gestational diabetes (GDM) has led to heterogeneity in the identification of GDM, thereby impacting the accurate estimation of the prevalence of GDM. We aimed to evaluate the effect of different diagnostic criteria for GDM on its prevalence among general populations of pregnant women worldwide, and also to investigate the prevalence of GDM based on various geographic regions.

**Methods:**

A comprehensive literature search was performed in PubMed, Scopus and Google-scholar databases for retrieving articles in English investigating the prevalence of GDM. All populations were classified to seven groups based-on their diagnostic criteria for GDM. Heterogeneous and non-heterogeneous results were analyzed using the fixed effect and random-effects inverse variance model for calculating the pooled effect. Publication bias was assessed by Begg’s test. The Meta-prop method was used for the pooled estimation of the prevalence of GDM. Meta-regression was conducted to explore the association between prevalence of GDM and its diagnostic criteria. Modified Newcastle–Ottawa Quality Assessment Scale for nonrandomized studies was used for quality assessment of the studies included; the ROBINS and the Cochrane Collaboration’s risk of bias assessment tools were used to evaluate the risk of bias.

**Results:**

We used data from 51 population-based studies, i.e. a study population of 5,349,476 pregnant women. Worldwide, the pooled overall-prevalence of GDM, regardless of type of screening threshold categories was 4.4%, (95% CI 4.3–4.4%). The pooled overall prevalence of GDM in the diagnostic threshold used in IADPSG criteria was 10.6% (95% CI 10.5–10.6%), which was the highest pooled prevalence of GDM among studies included. Meta-regression showed that the prevalence of GDM among studies that used the IADPSG criteria was significantly higher (6–11 fold) than other subgroups. The highest and lowest prevalence of GDM, regardless of screening criteria were reported in East-Asia and Australia (Pooled-P = 11.4%, 95% CI 11.1–11.7%) and (Pooled-P = 3.6%, 95% CI 3.6–3.7%), respectively.

**Conclusion:**

Over the past quarter century, the diagnosis of gestational diabetes has been changed several times; along with worldwide increasing trend of obesity and diabetes, reducing the threshold of GDM is associated with a significant increase in the incidence of GDM. The harm and benefit of reducing the threshold of diagnostic criteria on pregnancy outcomes, women’s psychological aspects, and health costs should be evaluated precisely.

**Electronic supplementary material:**

The online version of this article (10.1186/s13098-019-0406-1) contains supplementary material, which is available to authorized users.

## Background

Gestational diabetes mellitus (GDM), is one of the most common endocrinopathies during pregnancy which is defined as hyperglycemia at any time in pregnancy based on defined thresholds that are less than those considered for overt diabetes [1]. Placental production of diabetogenic hormones such as human placental lactogen in late pregnancy, leading to progressive insulin resistance; when adaptation β-cell hyperfunctionality during pregnancy fails to compensate maternal insulin resistance, it may lead to gestational diabetes [2, 3]. It is well documented that GDM is associated with adverse maternal and neonatal outcomes [4, 5] as well as lifelong risk of obesity and diabetes in both mother and child later in life [6, 7].

It is estimated that GDM affects around 7–10% of all pregnancies worldwide [8–11]; however the prevalence is difficult to estimate as rates differ between studies due to prevalence of different risk factors in the population, such as maternal age and BMI, prevalence of diabetes and ethnicity among women [12]. Moreover, screening strategies, testing methods and even diagnostic optimum glycemic thresholds for GDM remain the subject of considerable debate [13].

In this respect, the first definition of GDM was based on maternal risk for developing postpartum diabetes; subsequently, it was defined based on adverse maternal and neonatal outcomes [14]. The study of the Hyperglycaemia and Adverse Pregnancy Outcomes (HAPO) study [15] demonstrated a linear continuous correlation between increasing levels of maternal blood glucose levels on a 75-g oral glucose tolerance test (GTT) and adverse perinatal outcomes without specific threshold. In this respect, potential GDM diagnostic criteria were defined based on the odds ratio (OR) of 1.75, relative to the mean, for specific selected outcomes [15, 16].

In 2010, the International Association of Diabetes in Pregnancy Study Group (IADPSG) [17] endorsed 75-g oral glucose tolerance test, whereas in the United States and some countries GDM usually is screened and diagnosed based on the two-step screening strategy with a 3-h, 100-g OGTT after an abnormal 1-h, 50-g glucose challenge test (GCT). Furthermore, the World Health Organization (WHO) endorses the IADPSG diagnostic criteria for GDM, although the evidence for this recommendation was not very strong and was based on consensus. Nevertheless, this threshold, which was one of the lowest cut points for GDM diagnosis, has the high sensitivity and specificity [18].

However, the absence of evidenced-based and accepted ‘gold standards’ for the diagnosis of gestational diabetes as a screening strategy can lead to a heterogeneity in the identification of GDM in pregnant women [13] which may influence estimation of the prevalence of GDM and related health outcomes, as well as their health costs and quality of life.

The aim of this systematic review and meta-analysis hence was to evaluate the impact of different diagnostic criteria of blood glucose on the prevalence of GDM among general populations of pregnant women worldwide in different geographic regions.

## Methods

The ethics committee of the Research Institute for Endocrine Sciences, Shahid Beheshti University of Medical Sciences, approved this study.

This systematic review and meta-analysis was conducted based on the Preferred Reporting Items for Systematic Reviews and Meta-Analyses (PRISMA) [19] to assess the following objectives:

- To study the pooled prevalence of GDM among general population of pregnant women;
- To study the pooled prevalence of pregnant women based on the various diagnostic criteria of blood glucose;
- To study the pooled prevalence of pregnant women based on various GDM screening criteria groups of pregnant women in different geographic regions;
- To study the association between prevalence of GDM and its diagnostic criteria regardless of the geographic region.

### Search strategy

A comprehensive literature search was conducted in PubMed [including Medline], Web of Science, Google scholar and Scopus databases for retrieving original articles published in English language on the prevalence and incidence of gestational diabetes for all articles up to January 2018. Further, a manual search in the references list of studies included and other relevant reviews was used to maximize the identification of eligible studies. The following MeSH terms keywords, alone or in combination, were used for the search: “gestational diabetes” OR “gestational diabetes mellitus” OR “pregnancy induced diabetes” OR “gestational hyperglycemia,” OR “gestational glucose intolerance” AND “incidence” OR “prevalence” OR “epidemiology”.

### Selection criteria, study selection and data extraction

Studies were eligible if (I) they had population based design, (II) universally assessed the prevalence of GDM (III) and provided accurate screening strategies and thresholds of blood sugar in those screening test. We excluded non-original studies including reviews, commentaries, editorials, letters, meeting abstracts, case reports or any papers that did not provide accurate and clear data.

The screening of titles, abstracts and full-text articles was conducted independently by authors (SBG and MA), for determining final eligibility criteria. Disagreements were resolved through discussions with senior investigator (FRT). The general characteristics of the studies including “the first author name, journal, publication year, country of study, years of sampling, study design, sample size, population characteristics including age and BMI, PCOS definition, GDM screening strategy, GDM criteria and laboratory values of blood sugar tests, study quality assessment and prevalence of GDM were extracted from the studies included and assessed. To prevent extraction and data entry errors, a control check between the final data used in the meta-analysis and the original publications was performed by all authors.

### Study subgroups

To facilitate clinical interpretation of the results for statistically significant findings, all studies included were further classified to 7 groups based on the GDM screening strategy and the nearest threshold of blood sugar in the screening test as follows:

- Group 1 or IADPSG definition, screened based on OGTT with 75 g 2-h. Threshold: one value > 92, 180 and 153 mg/dL for fasting, 1, 2 and 3 h;
- Group 2, screened based on OGTT with 75 g 2-h. Threshold: one value > 100 and 144 mg/dL for fasting and 2 h;
- Group 3, screened based on OGTT with 75 g 2-h. Threshold: one value > 110 and 140 mg/dL for fasting, 1 and 2 h;
- Group 4, screened based on OGTT with 75 g 2-h. Threshold: value > 180 mg/dL for 2 h.
- Group 5, screened based on GCT with 50 g 1-h GCT, Threshold: values > 140 mg/dL following OGTT with 100 g 3-h. Threshold: two value > 95, 180, 155 and 140 mg/dL for fasting, 1, 2 and 3 h *or* GCT with 50 g 1-h GCT, Threshold: values > 140 mg/dL following OGTT with 75 g 3-h. Threshold: two values > 95, 180, 155 and 140 mmol/L for fasting, 1, 2 and 3 h;
- Group 6, screened based on Glucose challenge test (GCT) with 50 g 1-h, Threshold: 140 mg/dL following oral glucose tolerance test (OGTT) with 100 g 3-h. Threshold: two values > 105 or 190, 155, 165 and 145 mg/dL for fasting, 1, 2 and 3 h;
- Group 7, screened based on OGTT with 100 g 3-h. Threshold: one value > 120, 175, 155 and 140 mg/dL for fasting, 1, 2 and 3 h.

### Quality assessment and risk of bias

Quality of the studies was critically appraised for their methodology and results presentation. Two reviewers (SBG and MA) who were blinded to study author, journal name and institution evaluated the quality of the studies independently. The quality of observational studies was also assessed using the modification of the Newcastle–Ottawa Quality Assessment Scale for nonrandomized studies (NRS) [20] which evaluates the quality of published nonrandomized studies in terms of selection, comparability and outcomes. Studies with scores above 6 were considered as high quality, 3-5 as moderate and those with scores below than 3 as low quality.

We also evaluated risk of bias for studies included, using the ROBINS for NRS [21] and Cochrane Collaboration’s tool for assessing risk of bias for other methodological studies [22]. Five domains related to risk of bias were assessed in each cross-sectional study including: bias in assessment of exposure, bias in development of outcome of interest in case and controls, bias in selection of cases, bias in selection of controls, and bias in control of prognostic variable. In addition, 7 domains related to risk of bias were assessed bias in selection of exposed and non-exposed cohort, bias in assessment of exposure, bias in presence of outcome of interest at start of study, bias in control of prognostic variables, bias in the assessment of the presence or absence of prognostic factors, bias in the assessment of outcome, bias in adequacy regarding follow up of cohorts. Authors’ judgments were categorized as ‘‘low risk,’’ ‘‘high risk,’’ and ‘‘unclear risk’’ of bias (probably low or high risk of bias) [22].

### Statistical analysis

The software package STATA (version 12; STATA Inc., College Station, TX, USA) was applied to conduct statistical analysis. Heterogeneity between studies was assessed using I^2^ index and P > 0.05 was interpreted as heterogeneity. Heterogeneous and non-heterogeneous results were analyzed using the fixed effects and random-effects inverse variance models for calculating the pooled effect. Publication bias was assessed by Begg’s test. The Meta-prop method was used for pooled estimation of GDM prevalence. Meta-regression was conducted to explore the association between prevalence of GDM and its diagnostic criteria. In this respect, we used the HAPO definition criteria for screening with group 4 as the reference group for comparison.

In addition, meta-analysis of pooled prevalence of GDM was performed in the subgroups of some different geographical regions of countries, based on different GDM diagnostic classifications. P >  0.05 was set as significance level.

## Results

### Search results, study selection, study characteristics, and quality assessment

Additional file 1: Figure S1 illustrates the flow diagram of the search strategy and study selection. The search strategy yielded 3396 potentially relevant articles. According to the selection inclusion criteria, 338 articles were identified for further full-text assessment. Finally, we included 51 population-based studies which included data of 5,349,476 pregnant women for the meta-analysis. Table 1 presents the summary of studies assessing the prevalence of GDM.

**Table 1:** Summary of studies assessing GDM prevalence

Author, year	Country	Type of GDM screening test	GDM criteria	Year of sampling	Sample size	Prevalence of GDM	Quality scaling
Aljohani et al. 2008^a^	Canada	GCT with 50 g 1-h GCT, threshold: values above 7.8 mmol/L following OGTT with 100 g 3-h. Threshold: two value above 5.8, 10.6, 9.2 and 8.1 mmol/L for fasting, 1, 2 and 3 h	National criteria	1985–2004	324,605	2.9	Moderate
Al Mahroos et al. 2005^a^	Bahrain	GCT with 50 g 1-h GCT, threshold: values above 140 mg/dL following OGTT with 75 g 3-h. Threshold: two value above 95, 180, 155 and 140 mg/dL for fasting, 1, 2 and 3 h	Fourth international conference on GDM	2001–2002	10,495	13.3	High
Anna et al. 2008^b^	Australia	GCT with 50 g 1-h GCT, threshold: values above 7.8 mmol/L following OGTT with 75 g glucose. Threshold: value above 5.5, 8 mmol/L for fasting and 2 h	National criteria	1995–2005	950,737	3.7	High
Arora et al. 2015^b^	India	1. OGTT with 75 g glucose. Threshold: value above 5.1 and 8.5 mmol/L for fasting and 2 h 2. OGTT with 75 g glucose. Threshold: value above 7 and 7.8 mmol/L for fasting and 2 h	1. WHO 2013 2. WHO 1999	2009–2012	1. 5100 2. 5100	1. 34.9 2. 9	Moderate
Baptiste-Roberts et al. 2012^a^	USA	OGTT with 100 g 3-h. Threshold: value above 120 or 175, 155 and 140 mg/dL for fasting and 1 h, and did not return to normal in the 2- and 3-h	National criteria	1959–1966	28,358	1.7	High
Leng et al. 2015^a^	China	1. GCT with 50 g 1-h GCT, Threshold: values above 7.8 mmol/L following OGTT with 75 g 2-h. Threshold: one value above 5.1, 10.0 and 8.5 mg/dL for fasting, 1 and 2 h 2. GCT with 50 g 1-h GCT, Threshold: values above 7.8 mmol/L following OGTT with 75 g 2-h. Threshold: Fasting < 7.0 mmol/L and 2-h > 7.8 but < 11.1 mmol/L OR fasting > 6.1 but < 7.0 mmol/L and 2-h PG < 7.8 mmol/L	1. IADPSG 2. WHO1999	2010–2012	1. 17,808 2. 17,808	1. 7.7 2. 6.8	High
Chodick et al. 2010^a^	Israel	GCT with 50 g 1-h GCT, threshold: not mentioned, following OGTT with 100 g 3-h. Threshold: two value above 95, 180, 155 and 140 mg/dL for fasting, 1, 2 and 3 h	Carpenter and Coustan	1995–1999	185,416	6.07	High
Moses et al. 2011^a^	Australia	1. OGTT with 75 g glucose. Threshold: one value above or equal to 5.5 and 8.0 mmol/L for fasting and 2 h 2. OGTT with (not mentioned) g glucose. Threshold: one value above or equal to 5.1, 10.0 and 8.2 mmol/L for fasting, 1 and 2 h	1. ADIPS 2. IADPSG	NM*	1. 1275 2. 1275	1. 9.6 2. 13	Moderate
Erjavec et al. 2016^b^	Croatia	1. OGTT with 75 g glucose. Threshold: one value above or equal to 6.1 and 7.8 mmol/L for fasting and 2 h 2. OGTT with 75 g glucose. Threshold: one value above or equal to 5.1, 10.0 and 8.5 mmol/L for fasting, 1 and 2 h	1. WHO 1999 2. National criteria	1. 2010 2. 2014	1. 42,656 2. 39,092	1. 2.2 2. 4.7	High
Ferrara et al. 2004^a^	USA	1. GCT with 50 g 1-h, threshold: not mentioned, following OGTT with 100 g 3-h. Threshold: Two value above 95 or 180, 155 and 140 mg/dL for fasting, 1 and 2 h 2. 2 hpp > 200 mg/dL, 3. FBS > 126 mg/dL, 4. OGTT with 75 g 2-h, threshold: value above 140 mg/dL for 2 h, 5. GDM histort at time of hospital discharge	ADA, ACOG and WHO	1999–2000	267,051	6.33	Moderate
Ferrara et al. 2002^b^	USA	1. GCT with 50 g 1-h, threshold: 140 mg/dL following OGTT with 100 g 3-h. Threshold: two value above 105 or 190, 155, 165 and 145 mg/dL for fasting, 1, 2 and 3 h 2. GCT with 50 g 1-h, threshold: 140 mg/dL following OGTT with 100 g 3-h. Threshold: Two value above 95 or 180, 155, 140 and 145 mg/dL for fasting, 1, 2 and 3 h	1. NDDG 2. Carpenter and Coustan	1996	1. 26,481 2. 26,481	1. 3.2 2. 4.8	High
Gao et al. 2010^b^	China	(1) GCT with 50 g 1-h, Threshold: ≥ 7.8 mmol/L but < 11.1 mmol/L, (2) FPG ≥ 5.8 mmol/L, (3) Random FPG ≥ 5.8 mmol/L twice, following OGTT with 75 g 3-h. Threshold: two value above 5.3, 10.0, 8.6 and 7.8 mmol/L for fasting, 1, 2 and 3 h	ADA	2006	4179	17.9	Moderate
Hedderson et al. 2010^a^	USA	GCT with 50 g 1-h, threshold: not mentioned following OGTT with 100 g 3-h, threshold: two value above 95 or 180, 155, 140 and 145 mg/dL for fasting, 1, 2 and 3 h	ADA	1995–2004	216,089	5.8	High
Ignell et al. 2014^b^	Sweden	OGTT with 75 g glucose. Threshold: value above or equal 10.0 mmol/L for 2 h	European Association of the Study of Diabetes	2003–2012	156,144	2.2	Moderate
Jenum et al. 2012^a^	Norway	1. OGTT with 75 g glucose. Threshold: one value above or equal to 7 and 7.8 mmol/L for fasting and 2 h 2. OGTT with 75 g glucose. Threshold: one value above or equal to 5.1 and 8.5 mmol/L for fasting and 2 h	1. WHO 2. IADPSG	2008–2010	1. 759 2. 759	1. 13 2. 31.5	High
Ishak et al. 2003^a^	Australia	OGTT with 75 g glucose. Threshold: one value above or equal to 5.5 and 8 mmol/L for fasting and 2 h OR OGTT with 75 g glucose. Threshold: one value above or equal to 7.8 and 11 mmol/L for fasting and 2 h	National criteria	1988–1999	230,011	2.46	Moderate
Janghorbani et al. 2006^a^	UK	Random plasma glucose, threshold: 6.5 mmol/L following OGTT with 75 g glucose. Threshold: one value above or equal to 6 and 7.5 mmol/L for fasting and 2 h	WHO	1996–1997	4942	1.8	Moderate
Jesmin et al. 2014^b^	Bangladesh	1. GCT with 50 g 1-h, threshold: 7.8 mmol/L following OGTT with 75 g 2-h, threshold: ne value above or equal to 7 and 7.8 mmol/L for fasting and 2 h 2. GCT with 50 g 1-h, threshold: 7.8 mmol/L following OGTT with 75 g 2-h, threshold: ne value above or equal to 5.3 and 8.6 mmol/L for fasting and 2 h	1. WHO 2. ADA	2012–2013	1. 3447 2. 3447	1. 9.7 2. 12.9	Moderate
Kalamegham et al. 2010^a^	USA	GCT with 50 g 1-h, threshold: 130 mg/dL following OGTT with 100 g 3-h, threshold: ne value above or equal to 7 and 7.8 mmol/L for fasting and 2 h	ADA	2000–2007	18,307	8.6	Moderate
Lawrence et al. 2008^a^	USA	GCT with 50 g 1-h, threshold: not mentioned following (1) OGTT with 100 g 3-h, threshold: two value above or equal to 5.3, 10, 8.6 and 7.8 mmol/L for fasting, 1, 2 and 3 h OR (2) OGTT with 75 g glucose, threshold: two value above or equal to 5.3, 10 and 8.6 for fasting, 1 and 2 h OR (3) FBS ≥ 7 mmol/L OR (4) random plasma glucose ≥ 11.1 mmol/L	ADA	1999–2005	199,298	7.6	High
Leng et al. 2016^a^	China	GCT with 50 g 1-h, threshold: ≥ 7.8 mmol/L following OGTT with 75 g 2-h, threshold: value above 5.1, 10.0 and 8.5 mmol/L for fasting, 1 and 2 h	IADPSG	2010–2012	11,450	7.3	High
Magee et al. 1993^a^	USA	1. GCT with 50 g 1-h, threshold: ≥ 7.7 mmol/L following OGTT with 100 g 3-h, threshold: two value above 5.9, 10.6, 9.2 and 8.1 mmol/L for fasting, 1, 2 and 3 h 2. GCT with 50 g 1-h, threshold: ≥ 7.7 mmol/L following OGTT with 100 g 3-h, threshold: two value above 5.3, 10.1, 8.7 and 7.8 mmol/L for fasting, 1, 2 and 3 h	1. NDDG 2. Modified NDDG	1985–1986	1. 2019 2. 2019	1. 1.6 2. 5.8	High
McCarth et al. 2010^a^	Argentina	OGTT with 75 g glucose. Threshold: value above or equal to 7.8 mmol/L for 2 h	National criteria	NM*	1702	5.8	Moderate
Melchior et al. 2017^b^	Germany	GCT with 50 g 1-h, threshold: ≥ 135 and ≤ 200 mg/dL following OGTT with 75 g 2-h, threshold: value above 92, 180 and 153 mg/dL for fasting, 1 and 2 h	ICD-10	2014–2015	458,291	13.2	Moderate
Mizuno et al. 2016^b^	Japan	Random blood glucose, threshold: > 100 mg/dL following OGTT with 75 g 2-h, threshold: value above or equal to 92, 180 and 153 mg/dL for fasting, 1 and 2 h	National criteria	2011	8874	2.3	High
Murphy et al. 1993^a^	USA	GCT with 50 g 1-h, threshold: ≥ 7.8 mmol/L following OGTT with 75 g 2-h, threshold: value above 92, 180 and 153 mg/dL for fasting, 1 and 2 h	O’Sullivan criteria	1987–1988	605	5.8	Moderate
Lindqvist et al. 2014^b^	Sweden	OGTT with 75 g glucose. Threshold: value above or equal to 10 mmol/L for 2 h	European Association for the Study of Diabetes	2011–2012	20,822	2.2	High
Ostlund et al. 2003^a^	Sweden	OGTT with 75 g 2-h, threshold: value above or equal to 6.7 and 9 mmol/L for fasting and 2 h	WHO	1994–1996	4918	1.7	Moderate
O’Sullivan et al. 2011^a^	Ireland	1. OGTT with 75 g 2-h, threshold: value above 5.1, 10 and 8.5 mmol/L for fasting, 1 and 2 h 2. OGTT with 75 g 2-h, threshold: value above or equal to 7 and 11 mmol/L for fasting and 2 h	1. IADPSG 2. WHO	2006–2009	1. 5500 2. 5500	1. 12.4 2. 9.4	Moderate
Bhavadharini et al. 2016^b^	India	1. OGTT with 75 g 2-h, threshold: value above or equal to 5.1, 10 and 8.5 mmol/L for fasting, 1 and 2 h 2. OGTT with 75 g 2-h, threshold: value above or equal to 7.7 mmol/L for 2 h	1. IADPSG 2. WHO	2013–2014	1. 1774 2. 1774	1. 15.7 2. 10.5	High
Pu et al. 2015^a^	USA	OGTT with 100 g 3-h, threshold: Two value above 95, 180, 155 and 140 mg/dL for fasting, 1, 2 and 3 h	ICD-9	2007–2012	24,195	10.4	High
Sacks et al. 2012^a^	HAPO study	OGTT with 75 g 2-h, threshold: value above or equal to 5.1, 10.0 and 8.5 mmol/L for fasting, 1 and 2 h	IADPSG	2000–2006	23,957	17.8	High
Schmidt et al. 2001^a^	Brazil	1. OGTT with 75 g 2-h, threshold: value above or equal to 5.3, 10.0 and 8.6 mmol/L for fasting, 1 and 2 h 2. OGTT with 75 g 2-h, threshold: value above or equal to 7.0 and 7.8 mmol/L for fasting and 2 h	1. ADA 2. WHO	1991–1995	4977	1. 2.4 2. 7.2	High
Schmidt et al. 2000^a^	Brazil	OGTT with 75 g 2-h, threshold: value above or equal to 7.0 and 7.8 mmol/L for fasting and 2 h	WHO	1991–1995	5004	7.6	Moderate
Sella et al. 2013^a^	Israel	GCT with 50 g 1-h, threshold: not mentioned following OGTT with 100 g 3-h, threshold: two value above 5.3, 10.0, 8.6 and 7.8 mmol/L for fasting, 1, 2 and 3 h	Carpenter and Coustan criteria	2000–2010	367,247	3.6	High
Seshiah et al. 2007^a^	India	OGTT with 75 g 2-h, threshold: value above or equal to 140 mg/dL for 2 h	WHO	2007	4151	3.9	Moderate
Seshiah et al. 2008^a^	India	OGTT with 75 g 2-h, threshold: value above or equal to 140 mg/dL for 2 h	WHO	2005–2007	12,056	13.9	Moderate
Seyoum et al. 1999^a^	Ethiopia	OGTT with 75 g 2-h, threshold: value above or equal to 140 mg/dL for 2 h	WHO	1999	890	3.7	Moderate
Shand et al. 2008^b^	Australia	GCT with 50 g 1-h, threshold: value above or equal to 7.8 mmol/L following OGTT with 75 g 2-h, threshold: value above 5.5 and 8.0 mmol/L for fasting and 2 h	ADIPS	1998–2002	370,703	4.5	High
Sommer et al. 2014^a^	Norway	OGTT with 75 g 2-h, threshold: value above or equal to 5.1 and 8.5 mmol/L for fasting and 2 h	IADPSG	2008–2010	728	31.5	High
Sudasingh et al. 2016^b^	Sri Lanka	OGTT with 75 g 2-h, threshold: value above or equal to 126 and 140 mg/dL for fasting and 2 h	WHO	2014–2015	1600	12.1	Moderate
Tamayo et al. 2016^b^	Germany	GCT with 50 g 1-h, threshold: ≥ 135 mg/dL following OGTT with 75 g 2-h, threshold: value above 92, 180 and 153 mg/dL for fasting, 1 and 2 h	ICD-10	2013–2014	158,839	6.81	Moderate
Tan et al. 2017^a^	Australia	1. OGTT with 75 g 2-h, threshold: value above or equal to 5.5 and 8.0 mmol/L for fasting and 2 h 2. OGTT with 75 g 2-h, threshold: value above or equal to 5.1, 10 and 8.5 mmol/L for fasting, 1 and 2 h	IADPSG	2014–2015	2895	9	High
Trujillo et al. 2015^a^	Brazil	OGTT with 75 g 2-h, threshold: value above or equal to 92, 180 and 153 mg/dL for fasting, 1 and 2 h	IADPSG	1991–1995	4926	18	Moderate
Wahabi et al. 2017^2^	Saudi Arabia	OGTT with 75 g 2-h, threshold: value above or equal to 92–125, 180 and 153–199 mg/dL for fasting, 1 and 2 h	WHO	2013–2015	9723	24.2	Moderate
Wang et al. 2012^b^	USA	GCT with 50 g 1-h, threshold: value above or equal to 140 mg/dL following OGTT with 100 g 3-h, threshold: two value above 95, 180, 155 and 140 mg/dL for fasting, 1, 2 and 3 h	ADA	1997–2009	62,685	4.3	High
Xiong et al. 2001^a^	Canada	GCT with 50 g 1-h, threshold: value above or equal to 7.8 mmol/L following OGTT with 100 g 3-h, threshold: two value above 5.8, 10.5, 9.2 and 8 mmol/L for fasting, 1, 2 and 3 h	National criteria	1991–1997	111,563	2.5	Moderate
Yang et al. 2009^a^	China	GCT with 50 g 1-h, threshold: value above or equal to 7.9–11.0 mmol/L following OGTT with 75 g 2-h, threshold: two value above 5.3, 10.0 and 8.6 mmol/L for fasting, 1 and 2 h	ADA	2006	16,286	4.3	High
Yeung et al. 2017^a^	Canada	GCT with 50 g 1-h, threshold: value above or equal to 7.8 mmol/L following OGTT with 75 g 2-h, threshold: two value above 5.3, 10.6 and 8.9 mmol/L for fasting, 1 and 2 h *OR* following OGTT with 100 g 3-h, threshold: two value above 5.3, 10.0 8.6 and 7.8 mmol/L for fasting, 1, 2 and 3 h	ICD-10	2004–2010	498,013	6	High
Zhang et al. 2011^b^	China	GCT with 50 g 1-h, threshold: value above or equal to 7.8 mmol/L following OGTT with 75 g 2-h, threshold: two value above 6.1-7 and 7.8 mmol/L for fasting, 1 and 2 h	WHO	1999–2008	105,473	4.5	High
Zhu et al. 2017^a^	China	OGTT with 75 g 2-h, threshold: one value above 5.1, 10.6 and 8.5 mmol/L for fasting, 1 and 2 h	National criteria	2013	15,194	19.7	Moderate

* *NM* not mentioned

^a^Cohort study

^b^Cross sectional study

Details of the quality assessment of studies included are presented in Additional file 1: Tables S1, S2. Twenty-six studies were classified as high [16, 23–47], and 25 as moderate [8, 48–71]; no study had low quality. A total of 33.3% studies were cross-sectional and 66.6% were prospective or retrospective cohorts published between 1993 and 2017. Thirty-five studies were cohort [8, 16, 23, 25–27, 30–34, 38–40, 42, 43, 45, 46, 48, 50, 51, 54, 55, 57, 60–66, 69, 71, 72] and 16 cross-sectional [24, 28, 29, 35–37, 41, 44, 47, 49, 52, 53, 56, 67, 68, 70]. Fourteen (27.4%) studies, classified as group 1 [16, 33, 35, 37, 39, 42, 49, 59, 60, 62, 68–71] used IADPSG; 6 (11.7%) as group 2 [24, 41, 43, 47, 50, 54], 11 (21.5%) as group 3 [28, 31, 55–58, 63–67], 2 (3.9%) as group 4 [36, 53], 11 (21.5%) as group 5 [23, 27, 30, 32, 38, 40, 44–46, 51, 52], 4 (7.8%) as group 6 [8, 29, 34, 48] and 3 (5.8%) as group 7 [25, 26, 61].

In addition, 13 studies were conducted in the USA and Canada [8, 25, 29, 30, 32, 34, 38, 44, 46, 48, 51, 57, 60], five in Australia [24, 41, 43, 50, 54], seven in China and Japan [26, 33, 35, 45, 47, 52, 71], 9 in north Europe [31, 36, 42, 53, 55, 59, 61, 62, 68], six in India, Bangladesh and Sri Lanka [37, 49, 56, 64, 65, 67] and 10 were from other countries [23, 27, 28, 39, 40, 58, 63, 66, 69, 70], including Bahrain, Israel, Croatia, Argentina, Brazil, Ethiopia and Saudi Arabia. One study by the Hyperglycemia and Adverse Pregnancy Outcome (HAPO) Study Cooperative Research Group was originally performed in nine countries [16].

Considering the amount of literature included, except for USA, Canada and Australia, the most commonly used threshold in Asia and Europe was IADPSG. Australians were screened based on their national criteria (group 2). The most prevalent criteria used in USA and Canada was the method used for group 5.

### Meta-analysis and meta-regression of outcomes

Worldwide, the pooled overall prevalence of GDM among pregnant women, regardless of type of screening criteria categories was 4.4%, (Pooled overall P = 4.4%, 95% CI 4.3–4.4%). The overall pooled prevalence (95% CI) of GDM among different groups, depending on the diagnosis criteria used, is presented in Table 2. I^2^ index showed that except for subgroup 7, no significant heterogeneity were detected in the meta-analysis.

**Table 2:** Results of heterogeneity and publication bias estimation and subgroup meta-analysis for prevalence of gestational diabetes based on various GDM screening strategy group among pregnant women

	Sample size of participants	I^2^%	P value for Begg’s test	Pooled overall prevalence (95% CI)
GDM screening category^a^				
1	722,312	98	0.139	0.106 (0.105–0.106)
2	1,662,369	99	1.000	0.065 (0.057–0.072)
3	138,812	98	0.298	0.089 (0.071–0.107)
4	176,966	0	0.317	0.022 (0.022–0.023)
5	2,086,957	99	0.443	0.051 (0.051–0.051)
6	493,168	98	0.851	0.029 (0.028–0.029)
7	68,892	99	0.051	0.044 (0.013–0.074)
Overall	5,349,476	99	0.070	0.44 (0.043–0.044)

^a^Groups are defined as follows

Group 1 or HAPO definition who was screened based on OGTT with 75 g 2-h. Threshold: one value above 92, 180 and 153 mg/dL for fasting, 1, 2 and 3 h

Group 2 who was screened based on OGTT with 75 g 2-h. Threshold: one value above 100 and 144 mg/dL for fasting and 2 h

Group 3 who was screened based on OGTT with 75 g 2-h. Threshold: one value above 110 and 140 mg/dL for fasting, 1 and 2 h

Group 4 who was screened based on OGTT with 75 g 2-h. Threshold: value above 180 mg/dL for 2 h

Group 5 who was screened based on GCT with 50 g 1-h GCT, threshold: values above 140 mg/dL following OGTT with 100 g 3-h. Threshold: two value above 95, 180, 155 and 140 mg/dL for fasting, 1, 2 and 3 h *or* GCT with 50 g 1-h GCT, threshold: values above 140 mg/dL following OGTT with 75 g 3-h. Threshold: two value above 95, 180, 155 and 140 mmol/L for fasting, 1, 2 and 3 h

Group 6 who was screened based on glucose challenge test (GCT) with 50 g 1-h, Threshold: 140 mg/dL following oral glucose tolerance test (OGTT) with 100 g 3-h. Threshold: Two value above 105 or 190, 155, 165 and 145 mg/dL for fasting, 1, 2 and 3 h

Group 7 who was screened based on OGTT with 100 g 3-h. Threshold: one value above 120, 175, 155 and 140 mg/dL for fasting, 1, 2 and 3 h

**Fig. 1:**
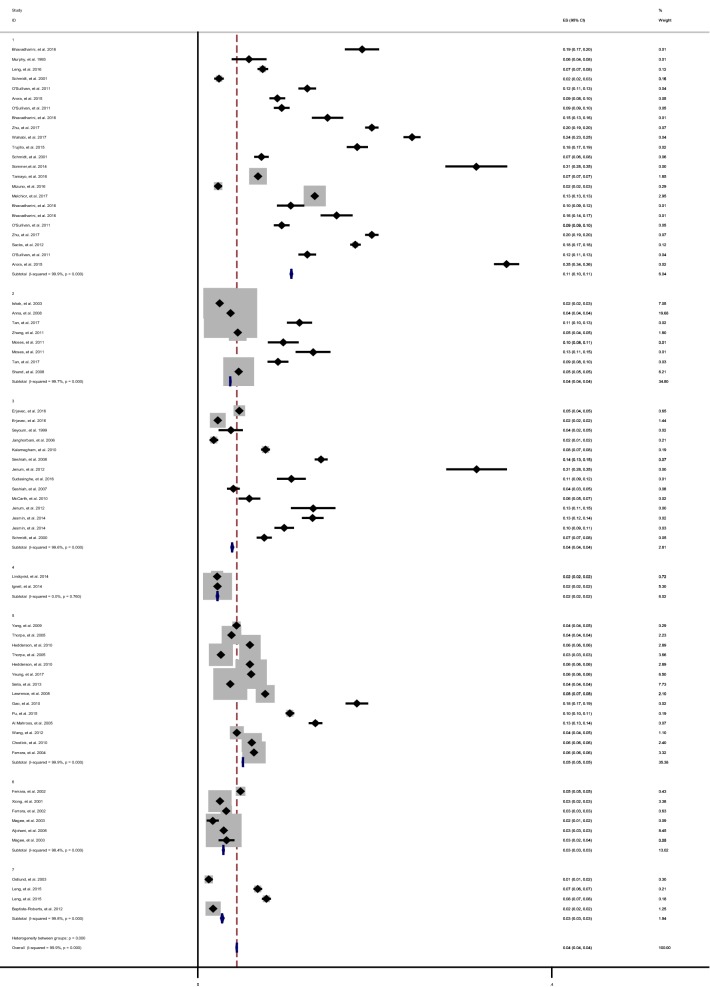
Forest plot of pooled Prevalence in subgroup of GDM diagnostic thresholds

The pooled prevalence of GDM in subgroup 1 was 10.6% (Pooled P = 10.6%, 95% CI 10.5–10.6%) which was the highest pooled prevalence of GDM among studies included. Moreover, the lowest prevalence of GDM was 2.2% in subgroup of 4 (Pooled overall P = 2.2%, 95% CI 2.2–2.3%) that used the cut of value of > 180 mg/dL for 2 h in OGTT-75 g glucose (Fig. 1). In this respect, the results of meta-regression showed that, exception for group 3, the prevalence of GDM among study that used the IADPSG criteria was significantly higher (6–11 fold) than other subgroups (Table 3) and (Additional file 1: Figure S2).

**Table 3:** Meta regression of the prevalence of GDM and GDM diagnostic threshold subgroups

GDM diagnostic criteria subgroups	Regression coefficient (95% CI)
2 vs. 1	− 0.06 (− 0.12, − 0.00)*
3 vs. 1	− 0.04 (− 0.09, 0.01)
4 vs. 1	− 0.11 (− 0.22, − 0.00)*
5 vs. 1	− 0.07 (− 0.12, − 0.021)*
6 vs. 1	− 0.11 (− 0.18, − 0.039)*
7 vs. 1	− 0.09 (− 0.17, − 0.01)*

Reference group: 1 (HAPO defined criteria)

* Statistically significant

Table 4 showed the pooled analysis of prevalence of GDM in various GDM screening criteria groups among pregnant women in different geographic regions. The highest and lowest prevalence of GDM, regardless of screening criteria, reported in East Asia and Australia was (Pooled P = 11.4%, 95% CI 11.1–11.7%) and (Pooled P = 3.6%, 95% CI 3.6–3.7%), respectively (Additional file 1: Figures S3–S7).

**Table 4:** Results of heterogeneity and publication bias estimation and subgroup meta-analysis for prevalence of gestational diabetes based on various GDM screening threshold group among pregnant women in different geographic regions

Regions	GDM diagnostic threshold subgroup	Number of studies included	Begg’s test P-value	I^2^%	Pooled measure of GDM (95% CI)
A	1	1	–	–	0.058 (0.039–0.076)
	2	–	–	–	–
	3	1	–	–	0.076 (0.072–0.080)
	4	–	–	–	–
	5	9	0.602	99	0.054 (0.054–0.054)
	6	6	0.851	98	0.029 (0.028–0.029)
	7	1	–	–	0.017 (0.016–0.019)
	Overall	18	0.692	99	0.045 (0.044–0.045)
B	1	6	0.850	99	0.152 (0.147–0.157)
	2	–	–	–	–
	3	5	0.625	99	0.094 (0.090–0.097)
	4	–	–	–	–
	5	–	–	–	–
	6	–	–	–	–
	7	–	–	–	–
	Overall	11	0.258	99	0.114 (0.111–0.117)
C	1	–	–	–	–
	2	7	0.625	99	0.036 (0.036–0.037)
	3	–	–	–	–
	4	–	–	–	–
	5	–	–	–	–
	6	–	–	–	–
	7	–	–	–	–
	Overall	7	0.625	99	0.036 (0.036–0.037)
D	1	4	0.090	99	0.078 (0.076–0.081)
	2	1	–	–	0.045 (0.044–0.046)
	3	–		–	–
	4	–		–	–
	5	2	0.317	99	0.053 (0.050–0.056)
	6	–	–	–	–
	7	2	0.317	91	0.072 (0.070–0.075)
	Overall	9	0.051	99	0.055 (0.054–0.056)
E	1	7	0.293	99	0.108 (0.107–0.108)
	2	–	–	–	–
	3	2	0.317	98	0.194 (0.175–0.213)
	4	2	0.317	0	0.022 (0.022–0.023)
	5	–	–	–	–
	6	–	–	–	–
	7	1	–	–	0.012 (0.009–0.015)
	Overall	12	0.520	100	0.060 (0.059–0.060)

A: USA and Canada; B: South Asia including India, Bangladesh and Sri Lanka; C: Australia; D: East Asia including China and Japan; E: north Europe including Finland, Ireland, Sweden, Norway and Germany

We performed a subgroup analysis based on the various threshold groups for screening in different geographic regions (Table 4). In this respect, the prevalence of GDM, based on the IADPSG criteria was (Pooled P = 15.2%, 95% CI 14.7–15.7%), (Pooled P = 7.8%, 95% CI 7.6–8.1%) and (Pooled overall P = 10.8, 95% CI 10.7–10.8%) respectively. USA, Canada and Australia did not use the IADPSG criteria most of the time. The pooled prevalence of GDM in USA and Canada, that mostly used criterion No. 5, were 5.4%; (Pooled P = 5.4%, 95% CI 5.4–5.4%) and in Australia screened based on criterion No. 2, was 3.6%, (Pooled P = 3.6%, 95% CI 3.6–3.7%). We did not have sufficient studies to perform meta-analyses in other regions.

### Publication bias and risk of bias

There was no substantial publication bias for meta-analyses based on the Begg’s test (Tables 2 and 4). Overall most of studies were judged as having low risk of bias for the evaluated domains; details are presented in Additional file 1: Figures S8, S9; as shown most cross-sectional and case–control studies had a low risk of bias in the assessment of exposure, development of outcome of interest in case and controls and selection of cases, approximately one-third of them had a high risk of bias in control of prognostic variables and selection of controls.

In addition, cohort studies had a low risk of bias for selection of exposed and non-exposed cohorts, assessment of exposure, presence of outcome of interest at start of study, outcome assessment, and adequacy of follow up of cohorts; however one-third of them had a high risk of bias in controlling prognostic variables and assessment of the presence or absence of prognostic factors and 3% of them had a high risk of bias in presence of outcome of interest at initiation of study.

## Discussion

The current meta-analysis of population based studies provided data on the impact of various thresholds of diagnostic GDM criteria on prevalence of GDM. Results of the meta-analysis showed that using lower glucose level thresholds as recommended by the IADPSG, identified significantly higher numbers (6–11 fold) of women with GDM, compared to other diagnostic criteria; in this respect, except for USA, Canada and Australia, this criteria was the most commonly used screening method worldwide. The highest prevalence of GDM was found in south Asia, where approximately 2 in ten women were diagnosed with GDM.

Despite the wide range of recommendations and guidelines for detection of women with GDM adopted by expert international societies [17, 73–80], there is strong controversy over the identification of GDM. Both the screening methods and diagnostic criteria vary among obstetricians and endocrine societies and more commonly even between regions within a single country. Screening approaches was include universal or targeted high risk screening, screening methods including fasting plasma glucose, random glucose and oral glucose challenge, diagnostic criteria including one steps or two, amount of the 75 g or 100 g glucose load, the duration of the test for 2 or 3 h, as well as the glucose threshold values, and whether 1 or 2 high glucose values are all used.

On the basis of the of Hyperglycemia and Adverse Pregnancy Outcome (HAPO) study [16], the International Association of Diabetes and Pregnancy Study Groups (IADPSG) suggested that a 75-g OGTT be performed and that GDM be diagnosed if any one of the following is observed: fasting plasma glucose > 92 mg/dL, 1 h: 180 mg/dL and 2 h: 153 mg/dL [17] selected based on the odds ratio of 1.75-fold, the mean for outcomes of the HAPO study. Although the IADPSG recommendations are the first evidence-based, large-scale guideline for GDM and are now widely used around the world, lack of sufficient data on the increased effectiveness in improving feto-maternal outcomes has led to the use of different criteria, which are often based on expert opinion and have all not been to acceptable universally.

However, the more stringent criteria of IADPSG, lead to higher prevalence of GDM among pregnant women and potentially increase the costs of care for many pregnant women worldwide [81]. Considering the fact that majority of births annually occur in low- and low–middle income countries with limited resources, the cost-effectivity of this definition must be precisely defined on short-term pregnancy and neonatal outcomes, as well as long-term cardio-metabolic benefits for mother and offspring and the cost effectiveness of treatment [82].

In addition, the diagnosis of GDM and its treatment is stressful situation can be accompanied by serious psychological challenges for women and their families due to the complex interaction between psychological factors based on patients experience [83, 84]. While not recognizing the GDM is associated with adverse pregnancy outcomes; over-diagnosis may leads to psychological stress, unnecessary treatments and impaired quality of life. Maternal concerns about one’s own and unborn health status may strong negative effects on the maternal health status, diminishing overall quality of life (QoL). Marchetti et al. in a systematic review, showed that QoL among women with GDM, is significantly worse in both the short and long term health status [72]. Moreover, a “diabetic” label carries familial and social stigma especially in gender biased cultures, possibly leading to conflict among families [83].

One of our main findings was the estimation of the prevalence of GDM worldwide. There are two documented meta-analyses that evaluated the prevalence of GDM; Eades et al. describes a meta-analysis of primary research data reporting the prevalence of gestational diabetes mellitus in the general pregnant population in Europe; they reported that the overall prevalence of GDM was 5.4% (95% CI 3.8–7.8%) [85]. In another recent meta-analysis, Nguyen et al. reported that the pooled prevalence of GDM in Eastern and Southeastern Asia was 10.1% (95% CI 6.5–15.7%), whereas those were across nations [9]. Results of both these studies are comparable with our meta-analysis. However, the first review was limited to developed countries in Europe which may have had a different prevalence of GDM from developing countries even in Europe. The second review were not references the population based studies and both of studies did not evaluate the effect of diagnostic criteria on GDM prevalence.

The present review has the strength of a large sample size with population-based design studies involving approximately five and a half million women, using different methods for screening and diagnosis of GDM and consistency of method, quality, and focus. However, there are some limitations that need to be considered when interpreting the results of this meta-analysis. This study focused on evaluating the prevalence of GDM based on different criteria and did not assess the impact of diagnostic criteria on maternal and neonatal outcomes, which is a limitation. In addition, most of the included studies did not report the maternal age and BMI; we could not adjust for these confounders in our analysis. Moreover, we included studies that used the universal screening strategy; so countries with a low prevalence, that mostly used the targeted high-risk screening strategy was not included in our meta-analysis, which may lead to overestimation of the prevalence of GDM in low prevalent areas e.g. north Europe. In addition, most of the included studies did not exclude the twin or multiple pregnancy in their report and some even reported the proportion of deliveries affected by GDM. However, since multiple pregnancies constitute approximately 3% of births [86, 87], it seems that could not confound the results. However, due to the lack of data available for some regions, we could not perform subgroup analysis in some areas. In addition, it should be noted that in the last quarter century, the definition of GDM has been changed several time. Moreover, the increasing trend of obesity and diabetes may increase the prevalence of gestational diabetes; and can lead to heterogeneity of data.

## Conclusion

Over the past quarter century, the diagnosis of gestational diabetes has been changed several times; there is still no general consensus about it. International communities have adopted different diagnostic methods and thresholds. Along with a worldwide increasing trend of obesity and diabetes, reducing the threshold for diagnosis of GDM are associated with a significant increase in the incidence of GDM. The harm and benefit of reducing the threshold of diagnostic criteria on pregnancy outcomes, women’s psychological aspects, and health costs should be evaluated precisely.

## Additional file

**Additional file 1.** **Table S1.** Quality assessment of studies included using the Newcastle–Ottawa Quality Assessment Scale for cohort studies. **Table S2.** Quality assessment of included studies using the Newcastle–Ottawa Quality Assessment Scale for cross-sectional study. **Figure S1.** Flow chart of the literature search for the systematic review and meta-analysis. **Figure S2.** Bubble plot of Prevalence GDM vs. GDM diagnostic criteria*. **Figure S3.** Forest plot of Pooled Prevalence for region A in subgroup of GDM diagnostic criteria. **Figure S4.** Forest plot of Pooled Prevalence for region B in subgroup of GDM diagnostic criteria. **Figure S5.** Forest plot of Pooled Prevalence for region C in subgroup of GDM diagnostic criteria. **Figure S6.** Forest plot of Pooled Prevalence for region D in subgroup of GDM diagnostic criteria. **Figure S7.** Forest plot of Pooled Prevalence for region E in subgroup of GDM diagnostic criteria. **Figure S8.** Risk of bias in cross-sectional studies. **Figure S9.** Risk of bias in cohort studies.

## Abbreviations

GDM: gestational diabetes mellitus
HAPO: hyperglycaemia and adverse pregnancy outcomes
OGTT: oral glucose tolerance test
OR: odds ratio
IADPSG: International Association of Diabetes in Pregnancy Study Group
WHO: World Health Organization
GCT: glucose challenge test
