# Supplementary material for: The impact of diagnostic criteria for gestational diabetes on its prevalence: a systematic review and meta-analysis
Source: Diabetol Metab Syndr. 2019 Feb 1;11:11. doi: 10.1186/s13098-019-0406-1 (PMC6359830; doi:10.1186/s13098-019-0406-1)

**Additional file**

Table S1. Quality assessment of studies included using the Newcastle–Ottawa Quality Assessment Scale for cohort studies

|  | **SELECTION** | | | | **COMPARABILITY** | **Outcome** | | | **Total scores** |
| --- | --- | --- | --- | --- | --- | --- | --- | --- | --- |
| **Author** | Representativeness of the exposed cohort | Selection of the non-exposed cohort | Ascertainment of exposure | No outcome of interest at start of study | A: Study controls for age and/or BMI  B: Study controls for other confounders | A: doctor’s diagnosis OR objective measurements  B: parent/self-reported doctor’s diagnosis OR use of medication | Follow-up long enough for outcomes ( at least 1 year) | Adequacy of follow up of cohorts |
| Aljohani (2008) (19) | * | - | * | * | - | * | * | * | 6 |
| Mahroos (2005) (13) | * | * | * | * | - | * | * | * | 7 |
| Baptiste-Roberts (2012) (35) | * | - | * | * | * | * | * | * | 7 |
| Leng (2015) (60) | * | * | * | * | * | * | * | * | 8 |
| Chodick (2010) (70) | * | - | * | * | ** | * | * | * | 8 |
| Moses (2011) (79) | * | - | * | * | - | * | * | * | 6 |
| Ferrara (2004) (108) | * | - | * | - | * | * | * | * | 6 |
| Hedderson (2010) (125) | * | * | * | * | * | * | * | * | 8 |
| Jenum (2012) (136) | * | * | * | * | ** | * | * | * | 9 |
| Ishak (2003) (137) | * | - | * | - | * | * | * | - | 5 |
| Janghorbani (2006) (143) | * | - | * | - | - | * | * | * | 5 |
| Kalamegham (2010) (149) | * | * | - | - | - | * | * | * | 5 |
| Lawrence (2008) (177) | * | - | * | * | ** | * | * | * | 8 |
| Leng (2016) (178) | * | * | * | * | * | * | * | * | 8 |
| Magee (2003) (185) | * | * | * | * | - | * | * | * | 7 |
| Murphy (1993) (203) | * | - | * | - | - | * | * | * | 5 |
| Ostlund (2003) (215) | * | - | - | - | - | * | * | * | 4 |
| O'Sullivan (2011) (218) | * | - | * | - | - | * | * | * | 5 |
| Pu (2015) (230) | * | - | * | * | * | * | * | * | 7 |
| Sacks (2012) (241) | * | * | * | * | - | * | * | * | 7 |
| Schmidt (2001) (247) | * | * | * | * | ** | * | * | * | 9 |
| Schmidt (2000) (248) | * | - | * | * | - | * | * | * | 6 |
| Sella (2013) (249) | * | * | * | - | * | * | * | * | 7 |
| Seshiah (2007) (251) | * | - | - | - | - | * | * | * | 4 |
| Seshiah (2008) (252) | * | - | * | * | - | * | * | * | 6 |
| Seyoum (1999) (254) | * | * | - | * | - | * | * | * | 6 |
| Sommer (2014) (267) | * | - | * | * | ** | * | * | * | 8 |
| Tan (2017) (275) | * | - | * | * | * | * | * | * | 7 |
| Trujillo (2015) (286) | * | * | * | * | - | * | * | * | 7 |
| Wahabi (2017) (292) | * | - | - | * | - | * | * | * | 5 |
| Xiong (2001) (305) | * | - | * | - | * | * | * | * | 6 |
| Yang (2009) (306) | * | * | * | * | ** | * | * | * | 9 |
| Yeung (2017) (309) | * | - | * | - | ** | * | * | * | 7 |
| Zhu (2017) (318) | * | - | - | - | ** | * | * | * | 6 |

Table S2. Quality assessment of included studies using the Newcastle–Ottawa Quality Assessment Scale for cross-sectional study.

|  | **SELECTION** | | | | **COMPARABILITY** | **Outcome** | | **Total scores** |
| --- | --- | --- | --- | --- | --- | --- | --- | --- |
| **Author** | Representativeness of the samples | Sample size | Non-responders | Ascertainment of the exposure (risk factor) | A: study controls for age and/or BMI  B: control for any additional factor | Assessment of the outcome  a) Independent blind assessment. **  b) Record linkage. **  c) Self report. * | Statistical test |
| Anna (2008) (24) | * | * | - | * | ** | ** | * | 8 |
| Arora (2015) (28) | * | * | - | * | - | ** | * | 6 |
| Bhavadhairini (2016) (47) | * | * | - | ** | ** | ** | * | 9 |
| Erjavec (2016) (101) | * | * | - | ** | ** | ** | * | 9 |
| Ferrara (2002) (110) | * | * | - | ** | * | ** | * | 8 |
| Gao (2010) (113) | * | * | - | ** | - | ** | - | 6 |
| Ignell (2014) (135) | * | * | - | ** | - | ** | - | 6 |
| Jesmin (2014) (146) | * | - | - | ** | - | ** | * | 6 |
| McCarth (2010) (187) | * | - | - | * | - | ** | * | 5 |
| Melchior (2017) (188) | * | * | * | * | - | * | * | 6 |
| Mizuno (2016) (192) | * | * | - | * | ** | * | * | 7 |
| Lindqvist (2014) (209) | * | * | - | * | ** | * | * | 7 |
| Shand (2008) (256) | * | * | - | * | * | ** | * | 7 |
| Sudasinghe (2016) (271) | * | - | - | * | - | ** | - | 4 |
| Tamayo (2016) (274) | * | * | - | - | - | ** | * | 5 |
| Wang (2012) (295) | * | * | - | * | * | ** | * | 7 |
| Zhang (2011) (315) | * | * | - | * | * | ** | * | 7 |

**Figure S1.** Flow chart of the literature search for the systematic review and meta-analysis.

Screening

Included

Eligibility

Identification

Records identified through database searches: (n = 3396)

(n = 2759)

Records remaining after duplicates removed: (n = 1575)

Records screened based on abstract: (n =944)

Full-text articles assessed for eligibility: (n = 338)

Total number of studies groups included in meta-analysis (n=51)

Figure S2: Bubble plot of Prevalence GDM vs. GDM diagnostic criteria*

* Reference group GDM diagnostic criteria= 1 (HAPO diagnostic criteria)

Figure S3: Forest plot of Pooled Prevalence for region A in subgroup of GDM diagnostic criteria

Figure S4: Forest plot of Pooled Prevalence for region B in subgroup of GDM diagnostic criteria

Figure S5: Forest plot of Pooled Prevalence for region C in subgroup of GDM diagnostic criteria

Figure S6: Forest plot of Pooled Prevalence for region D in subgroup of GDM diagnostic criteria

Figure S7: Forest plot of Pooled Prevalence for region E in subgroup of GDM diagnostic criteria

Figure S8: Risk of bias in cross-sectional studies.

A:

| Author, date | Bias in assessment of exposure (Risk factor) | Bias in development of outcome of interest in case and controls | Bias in selection of cases | Bias in selection of controls | Bias in control of prognostic variable (without case and control matching or adjustment in statistical methods ) |
| --- | --- | --- | --- | --- | --- |
| Anna (2008) (24) |  |  |  |  |  |
| Arora (2015) (28) |  |  |  |  |  |
| Bhavadhairini (2016) (47) |  |  |  |  |  |
| Erjavec (2016) (101) |  |  |  |  |  |
| Ferrera (2002) (110) |  |  |  |  |  |
| Gao (2010) (113) |  |  |  |  |  |
| Ignell (2014) (135) |  |  |  |  |  |
| Jesmin (2014) (146) |  |  |  |  |  |
| McCarth (2010) (187) |  |  |  |  |  |
| Melchior (2017) (188) |  |  |  |  |  |
| Mizuno (2016) (192) |  |  |  |  |  |
| Lindqvist (2014) (209) |  |  |  |  |  |
| Shand (2008) (256) |  |  |  |  |  |
| Sudasinghe (2016) (271) |  |  |  |  |  |
| Tamayo (2016) (274) |  |  |  |  |  |
| Wang (2012) (295) |  |  |  |  |  |
| Zhang (2011) (315) |  |  |  |  |  |
| Definitely No (low risk of bias) Probably no  Definitely yes (high risk of bias) Probably Yes | | | | | |

B:


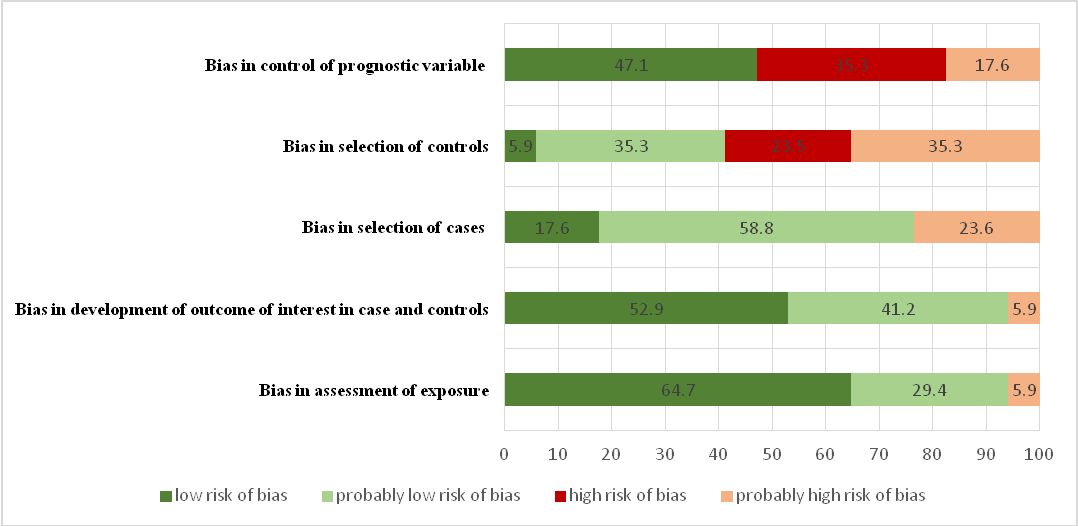


Figure S9: Risk of bias in cohort studies.

A:

| First Author, date | Bias in selection of exposed and non‐exposed cohorts | Bias in assessment of exposure | Bias in present of outcome of interest at start of study | Bias in control of prognostic variables (with matching or adjusting) | Bias in the assessment of the presence or absence of prognostic factors | Bias in the assessment of outcome | Bias in adequacy about follow up of cohorts |
| --- | --- | --- | --- | --- | --- | --- | --- |
| Aljohani (2008) (19) |  |  |  |  |  |  |  |
| Mahroos (2005) (13) |  |  |  |  |  |  |  |
| Baptiste-Roberts (2012) (35) |  |  |  |  |  |  |  |
| Leng (2015) (60) |  |  |  |  |  |  |  |
| Chodick (2010) (70) |  |  |  |  |  |  |  |
| Moses (2011) (79) |  |  |  |  |  |  |  |
| Ferrara (2004) (108) |  |  |  |  |  |  |  |
| Hedderson (2010) (125) |  |  |  |  |  |  |  |
| Jenum (2012) (136) |  |  |  |  |  |  |  |
| Ishak (2003) (137) |  |  |  |  |  |  |  |
| Janghorbani (2006) (143) |  |  |  |  |  |  |  |
| Kalamegham (2010) (149) |  |  |  |  |  |  |  |
| Lawrence (2008) (177) |  |  |  |  |  |  |  |
| Leng (2016) (178) |  |  |  |  |  |  |  |
| Magee (2003) (185) |  |  |  |  |  |  |  |
| Murphy (1993) (203) |  |  |  |  |  |  |  |
| Ostlund (2003) (215) |  |  |  |  |  |  |  |
| O'Sullivan (2011) (218) |  |  |  |  |  |  |  |
| Pu (2015) (230) |  |  |  |  |  |  |  |
| Sacks (2012) (241) |  |  |  |  |  |  |  |
| Schmidt (2001) (247) |  |  |  |  |  |  |  |
| Schmidt (2000) (248) |  |  |  |  |  |  |  |
| Sella (2013) (249) |  |  |  |  |  |  |  |
| Seshiah (2007) (251) |  |  |  |  |  |  |  |
| Seshiah (2008) (252) |  |  |  |  |  |  |  |
| Seyoum (1999) (254) |  |  |  |  |  |  |  |
| Sommer (2014) (267) |  |  |  |  |  |  |  |
| Tan (2017) (275) |  |  |  |  |  |  |  |
| Trujillo (2015) (286) |  |  |  |  |  |  |  |
| Wahabi (2017) (292) |  |  |  |  |  |  |  |
| Xiong (2001) (305) |  |  |  |  |  |  |  |
| Yang (2009) (306) |  |  |  |  |  |  |  |
| Yeung (2017) (309) |  |  |  |  |  |  |  |
| Zhu (2017) (318) |  |  |  |  |  |  |  |
| Definitely No (low risk of bias) Probably no  Definitely yes (high risk of bias) Probably Yes | | | | | | | |

B:


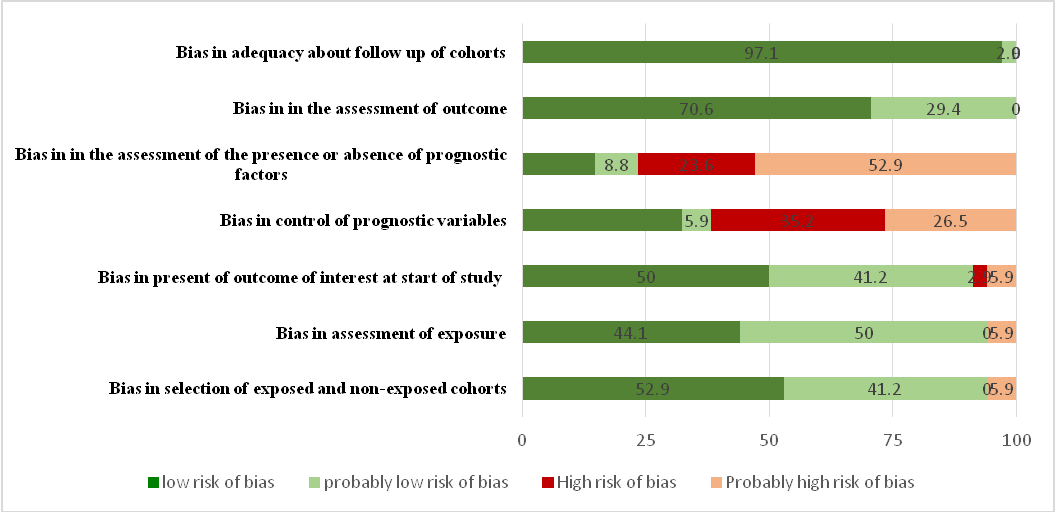

Supplement: Supplementary file 1 — Additional file 1. Table S1. Quality assessment of studies included using the Newcastle–Ottawa Quality Assessment Scale for cohort studies. Table S2. Quality assessment of included studies using the Newcastle–Ottawa Quality Assessment Scale for cross-sectional study. Figure S1. Flow chart of the literature search for the systematic review and meta-analysis. Figure S2. Bubble plot of Prevalence GDM vs. GDM diagnostic criteria*. Figure S3. Forest plot of Pooled Prevalence for region A in subgroup of GDM diagnostic criteria. Figure S4. Forest plot of Pooled Prevalence for region B in subgroup of GDM diagnostic criteria. Figure S5. Forest plot of Pooled Prevalence for region C in subgroup of GDM diagnostic criteria. Figure S6. Forest plot of Pooled Prevalence for region D in subgroup of GDM diagnostic criteria. Figure S7. Forest plot of Pooled Prevalence for region E in subgroup of GDM diagnostic criteria. Figure S8. Risk of bias in cross-sectional studies. Figure S9. Risk of bias in cohort studies. [file 13098_2019_406_MOESM1_ESM.doc]
